# Cerebrospinal fluid soluble TREM2 levels in frontotemporal dementia differ by genetic and pathological subgroup

**DOI:** 10.1186/s13195-018-0405-8

**Published:** 2018-08-16

**Authors:** Ione O. C. Woollacott, Jennifer M. Nicholas, Amanda Heslegrave, Carolin Heller, Martha S. Foiani, Katrina M. Dick, Lucy L. Russell, Ross W. Paterson, Ashvini Keshavan, Nick C. Fox, Jason D. Warren, Jonathan M. Schott, Henrik Zetterberg, Jonathan D. Rohrer

**Affiliations:** 1 Dementia Research Centre, Department of Neurodegenerative Disease, UCL Institute of Neurology, Queen Square, London, WC1N 3BG UK; 2 Department of Medical Statistics, London School of Hygiene and Tropical Medicine, London, UK; 3 Department of Molecular Neuroscience, UCL Institute of Neurology, Queen Square, London, UK; 4 UK Dementia Research Institute, London, UK; 5 Clinical Neurochemistry Laboratory, Sahlgrenska University Hospital, Mölndal, Sweden; 6 Department of Psychiatry and Neurochemistry, Institute of Neuroscience and Physiology, The Sahlgrenska Academy at the University of Gothenburg, Mölndal, Sweden

**Keywords:** Cerebrospinal fluid, Frontotemporal dementia, Microglia, Neuroinflammation, Progranulin, TREM2

## Abstract

**Background:**

Reliable biomarkers of frontotemporal dementia (FTD) are currently lacking. FTD may be associated with chronic immune dysfunction, microglial activation and raised inflammatory markers, particularly in progranulin (*GRN*) mutation carriers. Levels of soluble triggering receptor expressed on myeloid cells 2 (sTREM2) are elevated in Alzheimer’s disease (AD), but they have not been fully explored in FTD.

**Methods:**

We investigated whether cerebrospinal fluid (CSF) sTREM2 levels differ between FTD and controls, across different clinical and genetic subtypes of FTD, or between individuals with FTD due to AD versus non-AD pathology (based on CSF neurodegenerative biomarkers). We also assessed relationships between CSF sTREM2 and other CSF biomarkers (total tau [T-tau], tau phosphorylated at position threonine-181 [P-tau] and β-amyloid 1–42 [Aβ42]) and age and disease duration. Biomarker levels were measured using immunoassays in 17 healthy controls and 64 patients with FTD (behavioural variant FTD, *n* = 20; primary progressive aphasia, *n* = 44). Ten of 64 had familial FTD, with mutations in *GRN* (*n* = 3), *MAPT* (n = 4), or *C9orf72* (*n* = 3). Fifteen of 64 had neurodegenerative biomarkers consistent with AD pathology (11 of whom had logopenic variant PPA). Levels were compared using multivariable linear regressions.

**Results:**

CSF sTREM2 levels did not differ between FTD and controls or between clinical subgroups. However, *GRN* mutation carriers had higher levels than controls (mean ([SD] = 9.7 [2.9] vs. 6.8 [1.6] ng/ml; *P =* 0.028) and *MAPT* (3.9 [1.5] ng/ml; *P* = 0.003] or *C9orf72* [4.6 [1.8] ng/ml; *P* = 0.006) mutation carriers. Individuals with AD-like CSF had higher sTREM2 levels than those with non-AD-like CSF (9.0 [3.6] vs. 6.9 [3.0] ng/ml; *P* = 0.029). CSF sTREM2 levels were associated with T-tau levels in control and FTD groups and also with P-tau in those with FTD and AD-like CSF. CSF sTREM2 levels were influenced by both age and disease duration in FTD.

**Conclusions:**

Although CSF sTREM2 levels are not raised in FTD overall or in a particular clinical subtype of FTD, levels are raised in familial FTD associated with *GRN* mutations and in FTD syndromes due to AD pathology. Because CSF sTREM2 levels correlate with a marker of neuronal injury (T-tau), sTREM2 should be explored as a biomarker of disease intensity in future longitudinal studies of FTD.

## Background

Frontotemporal dementia (FTD) is a common cause of early-onset dementia, presenting with behavioural change (behavioural variant FTD [bvFTD]) or language impairment (primary progressive aphasia [PPA]). Around one-third of cases are familial, associated most commonly with mutations in progranulin (*GRN*), microtubule-associated protein tau (*MAPT*) or chromosome 9 open reading frame 72 (*C9orf72)* [1]. Pathologically, the majority of individuals have frontotemporal lobar degeneration (FTLD) with inclusions containing tau or transactive response DNA binding protein 43 (TDP-43), although some, particularly those with the logopenic variant of PPA (lvPPA), have Alzheimer’s disease (AD) pathology [2]. Reliable biomarkers that differentiate the pathological changes underlying sporadic FTD in vivo or that predict disease onset, severity or progression in sporadic and familial FTD are currently lacking. There is growing evidence that neuroinflammation and microglial dysfunction play a role in FTD, particularly in familial FTD secondary to *GRN* mutations [3, 4]. Inflammatory markers are variably altered in blood or cerebrospinal fluid (CSF) of patients with neurodegenerative disease, including across the clinical, genetic and pathological spectrum of FTD, and they could be useful as disease biomarkers in future clinical trials.

The protein triggering receptor expressed on myeloid cells 2 (TREM2) is an innate immune receptor expressed on microglia and on myeloid cells outside the brain [5, 6]. TREM2 is upregulated on activated microglia and involved in microglial phagocytosis [7–11], survival [12] and chemotaxis and response to neuronal injury [13]. Homozygous *TREM2* mutations lead to a rare syndrome called Nasu-Hakola disease [14], which is associated with an early-onset FTD-like dementia, and homozygous *TREM2* variants are associated with FTD-like syndromes without bony involvement [15–18]. TREM2 undergoes cleavage of its ectodomain to release a soluble TREM2 (sTREM2) fragment into the extracellular space [9], which is measurable in CSF and blood. Although raised CSF sTREM2 levels were initially described in neuroinflammatory conditions such as multiple sclerosis [19, 20], establishing the relationship between sTREM2 and other markers of disease has recently become of great interest in neurodegenerative disorders.

Most studies of CSF sTREM2 levels in dementia have focused on AD, but non-stratified patient cohorts have produced conflicting results, including increased [21, 22], reduced [9] or similar [23] levels in patients with AD compared with healthy controls. However, CSF sTREM2 levels may change according to disease stage in AD, with raised levels in mild cognitive impairment and early sporadic [24, 25] or pre-symptomatic familial [26] disease, but lower levels (similar to controls) in established disease [26]. This suggests that sTREM2 levels could be useful in tracking the disease course in AD or for determining proximity to disease onset in pre-symptomatic familial cases, and this may also be the case for other neurodegenerative diseases, such as FTD. CSF sTREM2 levels correlate with CSF levels of total tau (T-tau), a marker of neuronal injury, in AD cohorts, but generally not with β-amyloid 1–42 (Aβ42) levels [21, 22, 24–26]. This suggests that CSF sTREM2 may be a useful marker of microglial activation in response to neuronal injury, regardless of amyloid pathology, and hence worth exploring in FTD.

Previous studies of CSF sTREM2 levels in FTD have included small numbers of patients with undefined clinical subtypes and have found widely differing results, including lower [9], higher [22] or similar [21] levels in patients with FTD compared with healthy controls. It remains unclear whether CSF sTREM2 levels are altered in FTD or whether they differ between the various clinical subtypes of FTD. To our knowledge, no studies have compared CSF sTREM2 levels across groups of individuals with familial FTD to determine whether levels differ between the genetic subtypes of FTD. In addition, individuals may develop clinical syndromes consistent with FTD (bvFTD or PPA) due to underlying AD, rather than FTLD, pathology. It is unclear whether CSF sTREM2 levels differ in patients with similar clinical syndromes but contrasting pathologies.

Given the heterogeneous clinical, genetic and pathological nature of FTD and the urgent need for disease biomarkers, in this study we aimed to examine how CSF sTREM2 levels vary within a well-phenotyped cohort of symptomatic individuals with different clinical and genetic subtypes of FTD. We also aimed to clarify how CSF sTREM2 levels differ between individuals with clinical FTD syndromes due to AD versus FTLD pathology (as determined by their CSF neurodegenerative biomarker profile), as well as to establish whether sTREM2 levels correlate with levels of other CSF biomarkers previously explored in AD: T-tau, tau phosphorylated at position threonine 181 (P-tau) and Aβ42.

## Methods

### Participants

The cohort consisted of 64 individuals with dementia consistent with FTD who met consensus diagnostic criteria for either bvFTD [27] or PPA [28] and 17 cognitively normal controls. Individuals with dementia were recruited from the Specialist Cognitive Disorders Clinic at the National Hospital for Neurology and Neurosurgery, London, UK, or from the University College London (UCL) FTD cohort studies. Control participants were individuals with normal cognitive testing scores, normal neurological examinations and no underlying neurological conditions, recruited from cohort studies at UCL. Among the 64 individuals with FTD, there were 5 clinical subgroups: 20 had bvFTD, 16 non-fluent variant PPA (nfvPPA), 11 semantic variant PPA (svPPA), 14 lvPPA and 3 a PPA syndrome not otherwise specified (PPA-NOS; not fulfilling criteria of any of the other PPA phenotypes). All participants with FTD were genetically screened for all known FTD causative mutations, including the *C9orf72* expansion. Ten individuals had familial FTD, producing three genetic subgroups, due to mutations in *GRN* (*n* = 3), *MAPT* (*n* = 4) or *C9orf72* (n = 3). All familial cases had a clinical syndrome of bvFTD, except two individuals with *GRN* mutations who had nfvPPA. Demographics of the cohort are displayed in Table 1. Disease duration was calculated as the time, in years, between age at clinical onset of symptoms and date of CSF collection.

**Table 1:** Demographics and cerebrospinal fluid biomarker levels of control and dementia groups and all clinical subgroups

	Control	Dementia	bvFTD	nfvPPA	svPPA	lvPPA	PPA-NOS
No. of subjects	17	64	20	16	11	14	3
Male sex, *n* (% group)	6 (54.5)	45 (70.3)	19 (95.0)	9 (56.2)	7 (63.6)	7 (50.0)	3 (100.0)
Age at CSF collection, years, mean (SD)	63.7 (6.4)	64.6 (6.5)	63.4 (7.1)	66.9 (5.9)	60.8 (6.0)	66.5 (6.0)	64.6 (5.4)
Age at onset, years, mean (SD)	n/a	59.5 (6.9)	56.1 (6.7)	62.7 (6.1)	56.1 (5.3)	63.0 (6.7)	61.3 (4.1)
Disease duration at CSF collection, years, mean (SD); median (IQR)	n/a	5.1 (3.8); 4.2 (2.7–6.3)	7.4 (5.6); 6.3 (3.4–8.8)	4.2 (1.9); 4.2 (2.8–5.1)	4.7 (2.1); 4.6 (3.4–6.3)	3.5 (2.0); 3.1 (1.9–4.8)	3.2 (1.3); 2.7 (2.3–4.7)
CSF sTREM2, ng/ml, mean (SD)	6.8 (1.6)	7.4 (3.2)	6.3 (3.7)	7.8 (2.2)	7.4 (2.4)	8.2 (4.1)	8.7 (1.9)
CSF Aβ42, pg/ml, mean (SD)	1032.2 (214.3)	758.1 (280.7)	828.3 (171.7)	842.5 (298.0)	894.0 (247.4)	444.6 (148.3)	804.3 (434.2)
CSF T-tau, pg/ml, mean (SD)	332.6 (82.4)	531.4 (404.5)	351.9 (135.5)	490.6 (247.8)	395.5 (200.3)	968.8 (617.0)	403.3 (208.5)
CSF P-tau, pg/ml, mean (SD)	52.7 (10.6)	57.0 (30.6)	45.7 (17.0)	50.6 (18.1)	44.9 (19.8)	91.7 (41.6)	49.0 (14.4)
CSF T-tau/Aβ42 ratio, median (IQR)	0.3 (0.2–0.5)	0.5 (0.3–1.0)	0.4 (0.3–0.5)	0.6 (0.3–0.9)	0.4 (0.3–0.5)	2.0 (1.1–3.2)	0.4 (0.2–1.2)

*n/a* Not applicable; for others, *see* list of abbreviations

There was no difference in age at CSF collection between the dementia and control groups overall (mean difference − 0.87 years, unpaired *t* test: 95% CI − 4.41 to 2.67; *P* = 0.628), but the svPPA subgroup was younger than the nfvPPA subgroup (mean difference − 6.1 years, analysis of variance: 95% CI − 11.06 to − 1.13; *P* = 0.017) and lvPPA subgroup (mean difference − 5.7 years, 95% CI − 10.8 to − 0.6; *P* = 0.03). There was no significant difference in age between any of the genetic subgroups or compared with controls. There was a higher proportion of males in the dementia group than in the control group (70.3% vs. 54.5%; χ^2^ = 7.06, *df* = 1, *P* = 0.008) and a higher proportion of males in the bvFTD subgroup versus all other clinical subgroups and controls (χ^2^ = 17.5; *df* = 5, *P* = 0.004), other than the PPA-NOS group, in which all three participants were male (Table 1). There was no significant difference in disease duration between any of the clinical subgroups (Kruskal-Wallis test; *P* > 0.05).

### CSF collection, processing and biomarker analysis

For all participants, CSF was collected and stored using standardised procedures [29]. Briefly, samples were collected by lumbar puncture in polypropylene tubes, which were immediately transferred to the laboratory. Samples were centrifuged, and the supernatant was aliquoted and stored at − 80 °C within 30 minutes of arrival. CSF levels of T-tau, P-tau and Aβ42 were measured using commercially available INNOTEST sandwich enzyme-linked immunosorbent assays (Fujirebio Europe, Gent, Belgium).

### CSF sTREM2 immunoassay

CSF samples were analysed using an immunoassay protocol adapted from one published previously [9]. Streptavidin-coated 96-well plates (Meso Scale Discovery [MSD], Rockville, MD, USA) were blocked overnight at 4 °C in blocking buffer (0.5% bovine serum albumin [BSA] and 0.05% Tween 20 in PBS, pH 7.4). The plates were then incubated with the biotinylated polyclonal goat anti-human TREM2 capture antibody (0.25 μg/ml, BAF1828; R&D Systems, Minneapolis, MN, USA) diluted in blocking buffer, shaking for 1 hour at room temperature. They were subsequently washed five times with wash buffer (0.05% Tween 20 in PBS) and incubated for 2 hours shaking at room temperature with 50 μl per well of either (1) the standard curve constructed from recombinant human TREM2 protein (11084-H08H-50; Sino Biological Inc., Beijing, China) diluted in assay buffer (0.25% BSA and 0.05% Tween 20 in PBS, pH 7.4) to produce concentrations ranging between 4000 pg/ml and 62.5 pg/ml or (2) CSF samples diluted 1:4 in assay buffer. Standards and CSF samples were assayed in duplicate. Plates were again washed five times with wash buffer before incubation for 1 hour shaking at room temperature with the detection antibody, monoclonal mouse anti-human TREM2 antibody (1 μg/ml, [B-3]: sc373828; Santa Cruz Biotechnology, Dallas, TX, USA), diluted in blocking buffer. After five additional washing steps, plates were incubated with the secondary antibody (SULFO-TAG-labelled goat anti-mouse secondary antibody, R32AC-5; MSD) and incubated shaking for 1 hour in the dark. Last, plates were washed three times with washing buffer, then twice in PBS alone. The electrochemical signal was developed by adding MSD read buffer T 4× (R92TC-2; MSD) diluted 1:2, and the light emission was measured using the MSD Sector Imager 6000. The concentration of sTREM2 was calculated using a five-parameter logistic curve-fitting method with the MSD Workbench software package. Intra-assay coefficients of variation were less than 10%, and all samples were measured on the same day by a single operator using the same reagents.

### CSF AD biomarker classification

To examine whether CSF sTREM2 levels differ according to the underlying pathology in FTD, rather than by clinical syndrome, we used individuals’ CSF neurodegenerative biomarker profiles of T-tau and Aβ42 (Table 1) to classify all individuals with dementia into two pathological subgroups (AD biomarker-positive and AD biomarker-negative) based on whether there was a CSF biomarker profile consistent with AD (Table 2). We used a conservative cut-off of CSF T-tau/Aβ42 ratio > 1.0 as being consistent with dementia secondary to AD pathology, based on a previous study [29], and healthy controls were used as a comparison for both groups (all 17 controls had a CSF T-tau/Aβ42 ratio < 1.0). The AD biomarker-positive dementia subgroup consisted of 15 individuals with dementia, with a CSF T-tau/Aβ42 ratio > 1.0. As expected, the majority of these had lvPPA (*n* = 11); other diagnoses were nfvPPA (*n* = 2), svPPA (*n* = 1) and PPA-NOS (*n* = 1). The AD biomarker-negative subgroup contained the remaining 49 individuals with dementia with a CSF T-tau/Aβ42 ratio < 1.0. No significant difference in age at CSF was seen between the two biomarker subgroups and controls, but median disease duration at CSF was shorter in the AD biomarker-positive subgroup than in the AD biomarker-negative subgroup (2.9 versus 4.6 years; Mann-Whitney *U* test: *P* = 0.037). There were significantly more males in the AD biomarker-negative subgroup (73.4%) than in controls (54.5%) and the AD biomarker-positive (60.0%) subgroup (χ^2^ = 7.9, *df* = 2, *P* = 0.019).

**Table 2:** Demographics and cerebrospinal fluid biomarker levels of control group and cerebrospinal fluid Alzheimer’s disease biomarker-defined subgroups with dementia

	Control	AD biomarker-negative dementia (CSF T-tau/Aβ42 < 1.0)	AD biomarker-positive dementia (CSF T-tau/Aβ42 > 1.0)
No. of subjects	17	49	15
Male sex, *n* (% group)	6 (54.5)	36 (73.4)	9 (60.0)
Age at CSF, years, mean (SD)	63.7 (6.4)	64.1 (6.7)	65.9 (6.0)
Age at onset, years, mean (SD)	n/a	58.6 (6.8)	62.4 (6.7)
Disease duration at CSF collection, years, mean (SD); median (IQR)	n/a	5.6 (4.1); 4.6 (3.1–6.6)	3.5 (2.1); 2.9 (1.7–5.1)
CSF sTREM2, ng/ml, mean (SD)	6.8 (1.6)	6.9 (3.0)	9.0 (3.6)
CSF Aβ42, pg/ml, mean (SD)	1032.2 (214.3)	833.9 (265.4)	510.5 (165.7)
CSF T-tau, pg/ml, mean (SD)	332.6 (82.4)	373.5 (173.0)	1047.2 (511.3)
CSF P-tau, pg/ml, mean (SD)	52.7 (10.6)	44.8 (15.3)	97.0 (34.3)
CSF T-tau/Aβ42 ratio, median (IQR)	0.3 (0.2–0.5)	0.4 (0.3–0.7)	1.6 (1.2–3.2)

*AD* Alzheimer’s disease, *n/a* Not applicable; for others, *see* list of abbreviations

The dementia subgroups include individuals with FTD (bvFTD and PPA) defined by their CSF biomarker profile as stated

### Statistical analysis

CSF sTREM2 levels were first compared between the dementia group (all individuals with FTD, both bvFTD and PPA) and controls. The following subgroups were also compared: (1) clinical subgroups (bvFTD, nfvPPA, svPPA, lvPPA, PPA-NOS) with controls and between each subgroup; (2) genetic subgroups (*GRN*, *MAPT*, *C9orf72*) with controls and between each subgroup; and (3) pathological subgroups (AD biomarker-positive dementia and AD biomarker-negative dementia) compared with each other and with controls. We also examined associations between CSF sTREM2 levels and CSF T-tau, P-tau and Aβ42 levels in each group and subgroup. In addition, we determined whether CSF sTREM2 levels differed according to sex or were associated with other parameters that may influence biomarker levels (age and disease duration at CSF) in each group and subgroup. This guided adjustment for these variables in group and subgroup analyses. Associations of sTREM2 levels with age, disease duration and biomarker levels, and sex differences in sTREM2 levels, were not assessed within individual genetic subgroups, owing to small group size. All analyses were carried out using STATA14 (StataCorp, College Station, TX, USA), with a significance threshold of *P* < 0.05. Shapiro-Wilk tests were used to test assumptions of normality for all parameters.

Assessment of residuals in multivariable linear regression analyses of sTREM2 across groups revealed that these were normally distributed and so met the assumptions required for parametric linear regression analysis. Multivariable linear regressions were used to compare CSF sTREM2 levels between groups and subgroups, adjusting for age and sex in all analyses, and also for disease duration in analyses involving comparison of disease subgroups. Multivariable linear regressions were also used to investigate the association between sTREM2 levels and levels of CSF T-tau, P-tau and Aβ42 for each individual group and subgroup. These regressions were adjusted for age at CSF and sex (for the control group) and for age at CSF, sex and disease duration (for dementia and all subgroups). CSF sTREM2 levels (rather than the residuals of CSF sTREM2 levels as examined for regression analyses) were not normally distributed, so the Mann-Whitney *U* test was used to compare sTREM2 levels between males and females across the whole cohort and within each group and subgroup. Multivariable linear regression models were also used to assess associations between sTREM2 and age at CSF (adjusted for sex in the control group and both sex and disease duration in the dementia group and all subgroups) and disease duration at CSF (for dementia and all subgroups, adjusted for age and sex). Interactions between subgroup and age at CSF collection or disease duration were included for subgroup analyses to examine whether these associations differed by disease subgroup.

## Results

### CSF sTREM2 levels do not differ between FTD and controls or between clinical subtypes of FTD

**Fig. 1:**
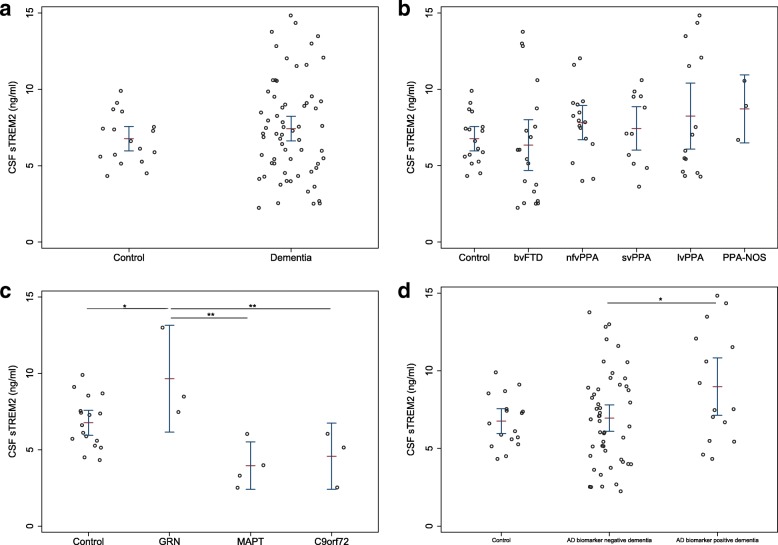
Comparison of CSF sTREM2 levels between groups and subgroups. Graphs show how CSF sTREM2 levels differ across (**a**) control and dementia (overall FTD, containing bvFTD and PPA) groups; (**b**) controls and clinical subgroups; (**c**) controls and genetic subgroups; and (**d**) controls and CSF biomarker-defined dementia subgroups (pathological subgroups). AD biomarker-negative dementia: CSF T-tau/Aβ42 ratio < 1.0; AD biomarker-positive dementia: CSF T-tau/Aβ42 ratio > 1.0. Horizontal bars show mean CSF sTREM2 levels and upper and lower 95% CIs for each group. *P < 0.05, **P < 0.01. *Aβ42* β-Amyloid 1–42, *AD* Alzheimer’s disease, *bvFTD* Behavioural variant frontotemporal dementia, *C9orf72* Chromosome 9 open reading frame 72 gene, *FTD* Frontotemporal dementia, *CSF* Cerebrospinal fluid, *GRN* Progranulin gene, *lvPPA* Logopenic variant primary progressive aphasia, *MAPT* Microtubule-associated protein tau gene, *nfvPPA* Non-fluent variant primary progressive aphasia, *PPA-NOS* Primary progressive aphasia not otherwise specified, *sTREM2* Soluble triggering receptor expressed on myeloid cells 2, *svPPA* Semantic variant primary progressive aphasia, *T-tau* Total tau

CSF sTREM2 levels did not significantly differ between individuals with dementia consistent with FTD (combined bvFTD and PPA) and controls (mean [SD] = 7.4 [3.2] versus 6.8 [1.6] ng/ml; *P* = 0.431) (Fig. 1a) or between any of the clinical subgroups and controls, adjusting for age and sex, or between any of the clinical subgroups, adjusting for age, sex and disease duration (Fig. 1b, Table 3). CSF sTREM2 values for control and dementia groups and for each clinical subgroup are summarised in Table 1. Detailed results of regression analyses comparing all groups and subgroups are presented in Table 3.

**Table 3:** Comparisons of cerebrospinal fluid soluble TREM2 levels between all disease groups and subgroups and controls

Groups compared	Mean (SEM) difference in CSF sTREM2 (ng/ml)	95% CI for CSF sTREM2 (ng/ml)	*P* value
Dementia vs. control	0.638 (0.806)	−0.966, 2.244	0.431
Clinical subgroups			
bvFTD vs. control	−0.349 (1.035)	−2.413, 1.713	0.736
nfvPPA vs. control	0.544 (1.009)	−1.468, 2.556	0.592
svPPA vs. control	1.145 (1.129)	−1.107, 3.397	0.314
lvPPA vs. control	1.040 (1.037)	−1.025, 3.106	0.319
PPA-NOS vs. control	1.840 (1.837)	−1.820, 5.502	0.320
nfvPPA vs. bvFTD	0.305 (1.153)	−2.004, 2.615	0.792
svPPA vs. bvFTD	1.344 (1.190)	−1.041, 3.729	0.264
lvPPA vs. bvFTD	0.723 (1.224)	−1.730, 3.176	0.557
PPA-NOS vs. bvFTD	1.228 (1.927)	−2.631, 5.088	0.526
svPPA vs. nfvPPA	1.038 (1.247)	−1.460, 3.537	0.409
lvPPA vs. nfvPPA	0.417 (1.101)	−1.789, 2.624	0.706
PPA-NOS vs. nfvPPA	0.922 (1.939)	−2.961, 4.806	0.636
lvPPA vs. svPPA	−0.621 (1.287)	−3.199, 1.957	0.631
PPA-NOS vs. svPPA	−0.116 (2.016)	−4.154, 3.923	0.954
PPA-NOS vs. lvPPA	0.505 (1.967)	−3.343, 4.445	0.798
Genetic subgroups			
*GRN* vs. control	2.748 (1.161)	0.331, 5.163	0.028^a^
*MAPT* vs. control	−2.230 (1.144)	−4.609, 0.149	0.065
*C9orf72* vs. control	−1.897 (1.266)	−4.529, 0.735	0.149
*GRN* vs. *MAPT*	4.978 (1.483)	1.893, 8.063	0.003^a^
*GRN* vs. *C9orf72*	4.644 (1.536)	1.448, 7.840	0.006^a^
*C9orf72* vs. *MAPT*	0.334 (1.408)	−2.595, 3.262	0.815
Pathological subgroups			
AD biomarker-positive dementia vs. controls	1.921 (0.996)	−0.062, 3.905	0.057
AD biomarker-negative dementia vs. controls	0.183 (0.817)	−1.446, 1.812	0.823
AD biomarker-positive dementia vs. AD biomarker-negative dementia	*F*(_2,58_) = 3.77, *P* = 0.029^a,b^		

*See* list of abbreviations for definition of abbreviations

Mean differences and 95% CIs are β values arising from multivariable linear regressions, adjusted for age and sex (disease groups and subgroups vs. controls), or age, sex and disease duration (between disease groups or subgroups, except for between genetic subgroups, which were adjusted for age and sex). Dementia group includes all individuals with FTD (bvFTD and PPA)

^a^Significant at *P* < 0.05

^b^Difference between groups changes with varying disease duration; results of regression analysis comparing groups overall are presented, rather than specific value for mean difference

### CSF sTREM2 levels are higher in *GRN* mutation carriers

There was a significant difference in sTREM2 levels across the genetic subgroups (*F*_3,21_ = 4.40, *P* = 0.015) (Fig. 1c, Table 3), adjusting for age and sex. The *GRN* mutation subgroup had higher levels than controls (mean [SD] = 9.7 [2.9] versus 6.8 [1.6] ng/ml; *P* = 0.028), and also than the *MAPT* (3.9 [1.5] ng/ml; *P* = 0.003) and *C9orf72* mutation (4.6 [1.8] ng/ml; *P* = 0.006) subgroups. There was no significant difference between other genetic subgroups or between *MAPT* or *C9orf72* subgroups and controls (Fig. 1c, Table 3).

### CSF sTREM2 levels are higher in FTD associated with AD pathology

In the pathological subgroup analysis, there was a trend towards a difference in CSF sTREM2 levels between the three groups (*F*_2,76_ = 2.55, *P* = 0.085), adjusting for age and sex. Levels were highest in the AD biomarker-positive dementia group (mean [SD] = 9.0 [3.6] ng/ml) compared with the other two groups (AD biomarker-negative dementia group = 6.9 [3.0] ng/ml; controls = 6.8 [1.6] ng/ml) (Fig. 1d). There was a significant difference in sTREM2 levels between the two pathological subgroups, with higher sTREM2 levels in those with positive AD biomarkers than in those with negative AD biomarkers (*F*_2,58_ = 3.77; *P* = 0.029), adjusting for age and sex. The difference in CSF sTREM2 levels between these two subgroups was affected by disease duration, with differences becoming more obvious as disease duration increased (Fig. 3c). A β value for the mean difference between these two subgroups is not presented in Table 3, because it would not be representative of this varying relationship. There was a trend towards higher levels in the AD biomarker-positive subgroup compared with controls (Fig. 1d), although this did not reach significance (β = 1.92; *P* = 0.057), and there was no difference between the AD biomarker-negative subgroup and controls (β = 0.183; *P* = 0.823).

### Associations between CSF sTREM2 and CSF T-tau, P-tau and Aβ42

**Fig. 2:**
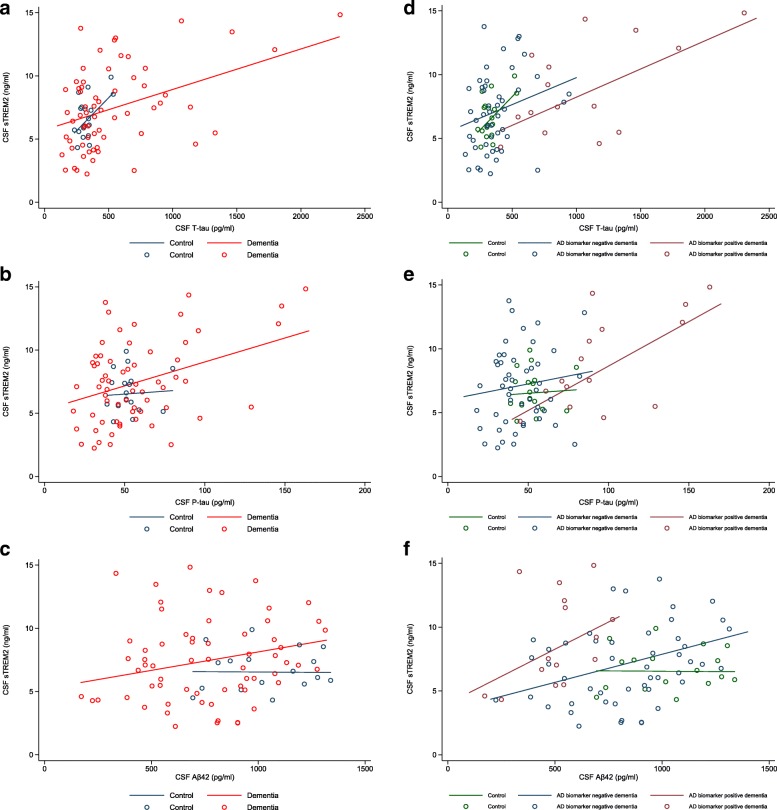
Relationship between CSF sTREM2 and CSF neurodegenerative biomarker levels. Graphs show associations between CSF sTREM2 and CSF T-tau (**a**), P-tau (**b**) and Aβ42 (**c**) levels for control and dementia groups, and between CSF sTREM2 levels and CSF T-tau (**d**), P-tau (**e**) and Aβ42 (**f**) levels for the control group and dementia subgroups defined by CSF biomarker status (pathological subgroups). AD biomarker-negative dementia: CSF T-tau/Aβ42 ratio < 1.0; AD biomarker-positive dementia: CSF T-tau/Aβ42 ratio > 1.0. Lines are group regression lines adjusted for age and sex (controls) and age, sex and disease duration (overall dementia group and biomarker-defined dementia subgroups). *See main text* for individual β and *P* values for each association. *Aβ42* β-Amyloid 1–42, *AD* Alzheimer’s disease, *CSF* Cerebrospinal fluid, *P-tau* tau phosphorylated at position threonine-181, *sTREM2* Soluble triggering receptor expressed on myeloid cells 2, *T-tau* Total tau

Associations between CSF sTREM2 levels and levels of CSF neurodegenerative biomarkers T-tau, P-tau and Aβ42 differed according to clinical diagnosis and CSF biomarker profile. In controls, CSF sTREM2 levels were positively associated with CSF T-tau levels adjusting for age and sex (β = 0.010, *P* = 0.033) (Fig. 2a), but not with P-tau (β = 0.009, *P* = 0.825) (Fig. 2b) or Aβ42 (β = − 0.0001, *P* = 0.958) (Fig. 2c) levels. However, in the dementia group, CSF sTREM2 levels were positively associated with levels of all three markers, after adjusting for age, sex and disease duration: T-tau (β = 0.003, *P* < 0.001) (Fig. 2a), P-tau (β = 0.038, *P* = 0.002) (Fig. 2b) and Aβ42 (β = 0.003, *P* = 0.033) (Fig. 2c). CSF sTREM2 levels were not associated with CSF T-tau, P-tau or Aβ42 in any of the clinical subgroups, except for lvPPA (most of whom had CSF consistent with AD), where they were positively associated with levels of all three markers: T-tau (β = 0.005, *P* < 0.001), P-tau (β = 0.075, *P* < 0.001) and Aβ42 (β = 0.015, *P* = 0.005). After stratifying the dementia group by CSF AD biomarker profile, there was an association between CSF sTREM2 and CSF T-tau in both pathological subgroups (AD biomarker-negative: β = 0.004, *P* = 0.049; AD biomarker-positive: β = 0.005, *P* = 0.002) (Fig. 2d), adjusting for age, sex and disease duration. There was also an association between sTREM2 and P-tau levels in the AD biomarker-positive dementia subgroup (β = 0.069, *P* = 0.004), but not in the AD biomarker-negative subgroup (β = 0.028, *P* = 0.284) (Fig. 2e), and, in contrast, with Aβ42 levels in the AD biomarker-negative subgroup (β = 0.005, *P* = 0.002), but not in the AD biomarker-positive subgroup (β = 0.009, *P* = 0.083) (Fig. 2f).

### CSF sTREM2 levels are influenced by age and disease duration

**Fig. 3:**
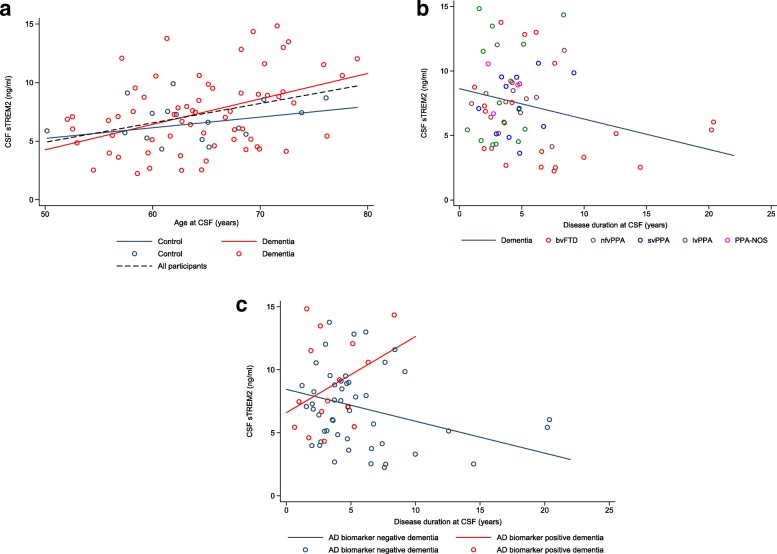
Relationship between CSF sTREM2 levels and age and disease duration at CSF collection. Graphs show CSF sTREM2 versus age (**a**) and disease duration (**b** and **c**) at CSF collection for the whole cohort (**a**, *dotted line*), dementia and control groups (**a** and **b**), and dementia subgroups defined by CSF biomarker status (**c**). Lines in (**a**) are group regression lines adjusted for sex (whole cohort and controls) and sex and disease duration (dementia group). Line in (**b**) is group regression line for dementia group adjusted for age and sex. Lines in (**c**) are group regression lines adjusted for age and sex for each CSF biomarker-defined dementia subgroup. AD biomarker-negative dementia: CSF T-tau/Aβ42 ratio < 1.0; AD biomarker-positive dementia: CSF T-tau/Aβ42 ratio > 1.0. *See main text* for individual β and *P* values for each association. *Aβ42* β-Amyloid 1–42, *AD* Alzheimer’s disease, *bvFTD* Behavioural variant frontotemporal dementia, *CSF* Cerebrospinal fluid, *lvPPA* Logopenic variant primary progressive aphasia, *nfvPPA* Non-fluent variant primary progressive aphasia, *PPA-NOS* Primary progressive aphasia not otherwise specified, *sTREM2* Soluble triggering receptor expressed on myeloid cells 2, *svPPA* Semantic variant primary progressive aphasia, *T-tau* Total tau

**Fig. 4:**
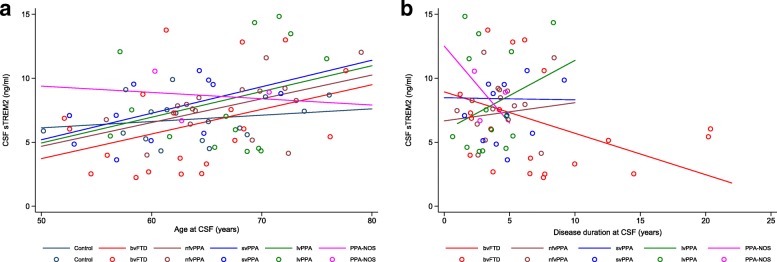
Relationship between CSF sTREM2 levels and age and disease duration at CSF collection in clinical subtypes of FTD. Graphs show CSF sTREM2 versus age (**a**) and disease duration (**b**) at CSF collection for each clinical subgroup. Lines in (**a**) are group regression lines adjusted for sex and disease duration; lines in (**b**) are group regression lines adjusted for age and sex. *See main text* for individual β and *P* values for each association. *bvFTD* Behavioural variant frontotemporal dementia, *FTD* Frontotemporal dementia, *CSF* Cerebrospinal fluid, *lvPPA* Logopenic variant primary progressive aphasia, *nfvPPA* Non-fluent variant primary progressive aphasia, *PPA-NOS* Primary progressive aphasia not otherwise specified, *sTREM2* Soluble triggering receptor expressed on myeloid cells 2, *svPPA* Semantic variant primary progressive aphasia

CSF sTREM2 levels were positively associated with age at CSF collection in the whole cohort (β = 0.165, *P* = 0.001) (Fig. 3a) and in individuals with dementia (β = 0.189, *P* < 0.001) (Fig. 3a). In the control group, this association was not significant (β = 0.061, *P* = 0.591) (Fig. 3a), but there was no significant difference between the dementia and control groups in the association between CSF sTREM2 levels and age (*P* = 0.312). In the clinical subgroup analysis, there was no significant difference between the coefficients for age by clinical subgroup (*P* = 0.964), and after adjusting for sex and disease duration, the association with age was generally similar in the majority of subgroups (Fig. 4a) (bvFTD β = 0.271, nfvPPA β = 0.184, svPPA β = 0.217, lvPPA β = 0.219, PPA-NOS β = 0.003), although this reached significance only for bvFTD (*P* = 0.017). In the pathological subgroup analysis, CSF sTREM2 levels were associated with age in both the AD biomarker-negative (β = 0.185, *P* = 0.006) and AD biomarker-positive (β = 0.282, *P* = 0.043) dementia subgroups. When we assessed interactions between pathological subgroup and age, there was no significant difference between the coefficients for age by subgroup (*P* = 0.528).

CSF sTREM2 levels were negatively associated with disease duration in the dementia group (β = − 0.235, *P* = 0.025) (Fig. 3b). In the clinical subgroup analysis, sTREM2 levels were negatively associated with disease duration in bvFTD (β = − 0.324, *P* = 0.014), but no significant association was seen in other clinical subgroups (Fig. 4b). Despite considerable heterogeneity in the slopes, there was no significant difference between the coefficients for disease duration by clinical subgroup (*P* = 0.275). In the pathological subgroup analysis, there was a significant difference in the coefficients for disease duration between the two dementia subgroups (*P* = 0.027). There was a significant negative association between CSF sTREM2 level and disease duration in the AD biomarker-negative subgroup (β = − 0.253, *P* = 0.018) but a trend towards a positive association in the AD biomarker positive subgroup (β = 0.604, *P* = 0.106). This resulted in the difference in CSF sTREM2 levels between these two subgroups increasing as disease duration increased (Fig. 3c).

CSF sTREM2 levels did not differ by sex within the whole cohort (median, males = 7.08 vs. females = 6.85 ng/ml; *P* = 0.992), or in controls, dementia, or any of the clinical or pathological subgroups, apart from in the svPPA subgroup, where males had higher sTREM2 levels than females (median = 9.51 vs. 4.98 ng/ml; *P* = 0.023).

## Discussion

This study shows that CSF sTREM2 levels do not differ overall between individuals with FTD and cognitively normal controls or between the various clinical subtypes of FTD. However, CSF sTREM2 levels are higher in those with familial FTD due to *GRN* mutations, albeit within a small cohort, and in individuals with a clinical syndrome consistent with FTD but CSF biomarkers consistent with underlying AD pathology. In addition, CSF sTREM2 levels are positively associated with levels of CSF T-tau in individuals with FTD and also with P-tau in individuals with likely AD pathology, and they are influenced by both age and disease duration.

Using a well-phenotyped cohort, we were able to compare CSF sTREM2 levels between individuals with FTD and controls, and across a variety of more distinct, clinically defined FTD syndromes, which, to our knowledge, has not been described previously. Other studies have found significantly lower [9] or higher [22] CSF sTREM2 levels in FTD than in controls. However, these studies assessed a much smaller number of FTD cases and without clearly defined clinical subgroups. There was significant variability in sTREM2 levels within our clinical subgroups, and large intergroup variability has been noted in studies of AD, where the substantial overlap between levels in each group limited the utility of sTREM2 to differentiate between AD and controls, despite a higher level in the AD group overall [21, 26]. CSF sTREM2 is also raised in a number of different neuroinflammatory [19, 20] and other neurodegenerative [21] diseases, limiting its diagnostic specificity for FTD. However, given the negative association with disease duration in our study, there may be a differential profile of CSF sTREM2 according to disease stage in FTD, as has been identified in the continuum from mild cognitive impairment to AD [24, 25], or according to disease intensity, as has been identified for serum and CSF neurofilament light chain levels in FTD [30, 31], which would be clinically useful.

We included a number of individuals with familial FTD in our study, enabling an exploratory analysis of differences in CSF sTREM2 between individuals with the three most common mutations linked to FTD (*GRN*, *MAPT* and *C9orf72*) and compared with cognitively normal controls. This adds to previous studies of raised inflammatory CSF markers in familial FTD, particularly in *GRN* mutation carriers [32–34]. Although we were able to include only a small number of individuals in each mutation group, we found that individuals with FTD due to *GRN* mutations had higher CSF sTREM2 levels than those with *MAPT* or *C9orf72* mutations, and compared with controls. This may be due to a link between *GRN* and *TREM2*, both of which are expressed by microglia, and thought to regulate microglial function and immune pathways in general. *GRN*-knockout mice have upregulated *TREM2* gene expression [3, 35] and excessive synaptic pruning mediated by aberrantly activated microglia [3]. sTREM2 promotes release of inflammatory cytokines and enhanced microglial activation and survival in mice [36]. Mouse models of homozygous *GRN* mutations and patients with heterozygous *GRN* mutations display excessive microglial activation on post-mortem brain tissue analysis [3, 37–40] and dysregulated levels of other inflammatory markers [32–34, 41]. Increased microglial activation in the context of *GRN* haploinsufficiency could lead to enhanced *TREM2* expression by microglia, increasing release of sTREM2 into the CSF and promoting survival of dysfunctional microglia or exacerbating neuronal damage through excessive phagocytosis.

Although downregulating *TREM2* expression or reducing CSF sTREM2 levels could be a therapeutic target for individuals with FTD secondary to *GRN* mutations, animal models of multiple sclerosis deteriorated with TREM2 inhibition [42]. In addition, the homozygous *TREM2* mutations that cause the frontal lobe dementia associated with Nasu-Hakola disease impair TREM2 function, locking microglia in a homeostatic, rather than phagocytic, state [10] and produce very low CSF sTREM2 levels [9]. This suggests that TREM2 may be protective in some circumstances. There is clearly a fine balance between TREM2 activity and suppression. However, CSF sTREM2 levels could rise in pre-symptomatic *GRN* mutation carriers before symptom onset and act as a useful marker of disease proximity, as in familial AD [26].

Because several studies have shown higher CSF sTREM2 levels in amnestic AD than in controls [21, 22, 24], we hypothesised that individuals with a clinical diagnosis of an FTD syndrome (i.e., bvFTD or PPA) but underlying AD pathology would have higher sTREM2 levels than controls. We were also keen to establish whether sTREM2 levels differ by underlying pathology (AD versus FTLD) in those with FTD. This is of particular use in the FTD field because certain patients, particularly those with lvPPA, may have underlying AD pathology and differing relationships between CSF sTREM2 and other disease biomarkers compared with individuals with FTLD, which could prove an issue for future clinical trials. By stratifying individuals with FTD by their CSF neurodegenerative biomarker profile (into AD biomarker-positive and AD biomarker-negative groups), we were able compare CSF sTREM2 levels (and relationships with other biomarkers) between biochemically defined, rather than clinically defined, syndromes. The significantly higher CSF sTREM2 levels in individuals with FTD but AD-like CSF (AD biomarker-positive group) than in those with non-AD-like CSF (AD biomarker-negative group), who most likely have FTLD, in our study suggests that individuals with significant neuronal injury due to AD (combined tau and amyloid pathology) may have more microglial activation and sTREM2 release into the CSF than those with FTLD. This is supported by our finding that CSF sTREM2 levels did not significantly differ between individuals with FTD and non-AD-like CSF and controls. There was a trend towards higher CSF sTREM2 levels in those with FTD and AD-like CSF than controls, although this did not reach significance, perhaps due to small group size. Interestingly, the difference in CSF sTREM2 levels between individuals with likely underlying AD versus FTLD became more pronounced with increasing disease duration. This most likely occurred because CSF sTREM2 levels were negatively associated with disease duration in those with FTLD, and there was a trend towards a positive association with disease duration in those with AD. In individuals with FTLD, microglial activation could decrease more over time, leading to a gradual decline in CSF sTREM2 levels. Alternatively, this separation may have occurred because several individuals with FTD and non-AD-like CSF had much longer disease durations (perhaps due to a less intense disease process) and much lower sTREM2 levels than the majority of those with AD-like CSF. Longitudinal CSF data from both groups are required to examine this further.

We went on to explore the relationship between levels of CSF sTREM2 and validated neurodegenerative biomarkers that reflect neuronal injury (T-tau), hyperphosphorylated tau (P-tau) and amyloid pathology (Aβ42). In individuals with FTD but likely AD pathology, higher CSF sTREM2 levels were associated with higher CSF T-tau and P-tau levels. This is consistent with associations between CSF sTREM2 and T-tau and/or P-tau levels in amnestic AD [21, 24, 25], as well as in our lvPPA subgroup, the majority of whom had AD-like CSF. We found that CSF sTREM2 levels were associated with T-tau (but not P-tau) levels in those with FTD due to likely FTLD, consistent with the theory that sTREM2 levels may also rise in the context of neuronal injury without concurrent hyperphosphorylated tau or amyloid pathology [22, 24–26]. In our control group, higher CSF sTREM2 levels were associated with higher CSF T-tau levels, but not with P-tau or Aβ42. Other studies have found associations with levels of T-tau, P-tau, or all three markers (T-tau, P-tau and Aβ42) in healthy controls [23–26]. This may reflect differential effects of microglial activation in response to mild age-related neuronal injury between individual cohorts, the variability of CSF T-tau and P-tau levels in healthy ageing [43, 44], or the variety of control group age distributions across studies. Surprisingly, we found a small but significant positive association between CSF sTREM2 and Aβ42 levels in FTD overall and in those with FTD and likely FTLD (rather than AD) pathology. This association with Aβ42 was observed in the control groups of three other studies [23–25], one of which speculated that the positive correlation between sTREM2 and Aβ42 levels was due to very early pre-symptomatic AD in some control individuals, because CSF Aβ42 may transiently increase due to reduced clearance before it decreases [23]. The positive association in our overall FTD group likely reflects that some individuals in this group were in a different stage of the AD pathology continuum than others, particularly because it included individuals with lvPPA. It remains unclear why there was also a positive association with Aβ42 in individuals with likely FTLD, although there may well be reduced clearance of amyloid in the context of extensive other pathology. However, overall in FTD there appears to be more of an association between CSF sTREM2 and T-tau and P-tau levels, than with Aβ42 levels, suggestive of a stronger link between sTREM2 and neuronal injury, than amyloid pathology itself. This is consistent with previous studies in AD [21–26].

We also examined relationships between sTREM2 levels and relevant clinical parameters such as age, disease duration and sex, which may affect TREM2 expression. The positive association between CSF sTREM2 levels and age in FTD is consistent with studies of AD [22–25]. Increased microglial activity associated with ageing leads to increased microglial TREM2 expression in healthy individuals [45] and in AD [46], and this could also be the case in FTD. We did not find a significant association with age in controls, as has been found previously [23, 25], perhaps because our control group was generally younger (to match to the FTD group) and smaller than those in other studies. Other studies’ control groups had a broader age range and so may have included some older, cognitively normal individuals with early AD pathology (and higher sTREM2 levels), leading to an apparently positive association between sTREM2 levels and age. We would suggest that future studies of CSF sTREM2 levels in neurodegenerative disease cohorts explore associations with age and consider adjusting group analyses for age at CSF collection.

Because previous research has shown that CSF sTREM2 levels vary by disease stage in mild cognitive impairment and AD [24, 25], we examined the relationship between CSF sTREM2 levels and disease duration in FTD. CSF sTREM2 levels were negatively associated with disease duration in FTD overall and particularly in bvFTD, where the widest range of disease durations was present. In the early stages of FTD, CSF sTREM2 levels may be high (due to florid microglial activation in response to incipient neurodegeneration), whereas later on in disease, levels may decrease, perhaps as compensatory microglial overactivation is overwhelmed by neurodegeneration. It has been postulated that this may occur in AD, and CSF sTREM2 levels could therefore act as a marker of disease progression [24]. An alternative explanation for our findings is that CSF sTREM2 levels may just be lower in individuals with FTD who have less aggressive disease and hence longer disease durations. CSF sTREM2 levels could therefore be a biomarker of rate of neuronal injury and disease intensity in FTD, in keeping with the positive association with CSF T-tau levels in individuals with non-AD-like CSF, who most likely have FTLD. Although the lack of longitudinal data in our study precludes a conclusion that levels of CSF sTREM2 rise and then fall over the disease course in FTD, our findings emphasise the importance of future studies assessing associations between CSF biomarker levels and disease duration.

Although CSF sTREM2 levels did not differ by sex within our whole cohort or in our dementia or control groups, there was a difference in the svPPA subgroup, with higher CSF sTREM2 levels in males. The majority of studies focusing on AD have not found an association between CSF sTREM2 levels and sex [21, 23–25], although significantly higher levels [22] or a trend towards higher levels [26] have been observed in males. Although it remains unclear whether sex affects sTREM2, we adjusted all analyses for sex.

A limitation of our study is that some of the FTD clinical and genetic subgroups were rather small, which may have limited our power to detect significant differences between groups. However, this is inherent to a disease such as FTD, where rarer subtypes exist, and it is difficult to avoid when analysing biomarker levels across a broad clinical and pathological spectrum of disease and when employing CSF collection and biomarker analysis at one centre in order to minimise inter-centre variation. Other studies with multi-centre CSF sources have shown significant variability in sTREM2 levels between centres [25], which we were keen to avoid. Our dementia group contained individuals with a diagnosis of an FTD syndrome, including those typically associated with FTLD-TDP or FTLD-tau (bvFTD, svPPA and nfvPPA) and those associated with AD pathology (lvPPA), which in theory could have differentially affected sTREM2 levels within the group as a whole. However, we were able to dissect out any differences in sTREM2 linked to differing pathologies through stratification of all patients with dementia by their CSF biomarker profile. Although most cases of dementia were not pathologically confirmed, all met recent diagnostic criteria for bvFTD [27] or PPA [28], and our CSF ratio cut-off was intentionally stringent to minimise misclassification of cases into the wrong pathology subgroup. Our dementia group combined individuals with a wide range of disease durations, which we showed was independently associated with sTREM2 levels. However, we adjusted analyses for disease duration wherever possible to account for this. We did not include any individuals with mild cognitive impairment, because this is typically a ‘pre-AD’ rather than a ‘pre-FTD’ state, nor did we analyse longitudinal CSF samples or samples from pre-symptomatic mutation carriers at risk of familial FTD. This means we cannot definitively conclude whether CSF sTREM2 levels change over the disease course, and therefore reflect disease proximity, intensity or progression in FTD, or how sTREM2 relates to changes in other CSF biomarkers such as T-tau over time.

## Conclusions

Although CSF sTREM2 does not seem useful for differentiating between individuals with FTD and healthy controls, or for delineating a particular clinical subtype of FTD, levels are higher in familial FTD associated with *GRN* mutations (albeit within a small preliminary cohort) and in individuals with a clinical syndrome consistent with FTD but underlying AD, rather than FTLD, pathology. Because CSF sTREM2 levels correlate with a measure of neuronal injury (T-tau), they may reflect disease intensity in FTD, but this requires further exploration.

Future studies should analyse CSF sTREM2 levels within larger cohorts of individuals with FTD, across a variety of clearly defined clinical subgroups, and ideally in pathologically confirmed cases. Inclusion of a larger number of familial FTD cases with mutations in *GRN*, *MAPT* and *C9orf72* (which have known pathology) would be helpful in this regard and would enable confirmation of our preliminary observations of higher levels in symptomatic *GRN* mutation carriers. Assessment of CSF sTREM2 levels in pre-symptomatic individuals at risk of familial FTD could establish if and when levels change prior to expected symptom onset. This would help to elucidate whether CSF sTREM2 levels may be useful as a biomarker of disease proximity in FTD, which, if validated, may be useful for guiding timely initiation of treatments or assessing treatment response in clinical trials. This would maximise the chance of benefitting individuals before significant neurodegeneration occurs. Exploration of relationships between baseline and longitudinal measurements of CSF sTREM2 levels and other markers of disease intensity (such as serum or CSF neurofilament light levels or frontal lobe atrophy rate) would also enable determination of whether CSF sTREM2 can be used as a biomarker of disease intensity in sporadic or familial FTD.

## Abbreviations

Aβ42: β-Amyloid 1–42
AD: Alzheimer’s disease
BSA: Bovine serum albumin
bvFTD: Behavioural variant frontotemporal dementia
*C9orf72*: Chromosome 9 open reading frame 72 (gene)
CSF: Cerebrospinal fluid
FTD: Frontotemporal dementia
FTLD: Frontotemporal lobar degeneration
*GRN*: Progranulin (gene)
lvPPA: Logopenic variant primary progressive aphasia
*MAPT*: Microtubule-associated protein tau (gene)
nfvPPA: Non-fluent variant primary progressive aphasia
PPA: Primary progressive aphasia
PPA-NOS: Primary progressive aphasia not otherwise specified
P-tau: tau phosphorylated at position threonine-181
sTREM2: soluble triggering receptor expressed on myeloid cells 2
svPPA: Semantic variant primary progressive aphasia
TDP-43: Transactive DNA response binding protein 43
TREM2: Triggering receptor expressed on myeloid cells 2
T-tau: Total tau

## References

[1] Woollacott IOC, Rohrer JD (2016). The clinical spectrum of sporadic and familial forms of frontotemporal dementia. J Neurochem.

[2] Lashley T, Rohrer JD, Mead S, Revesz T (2015). Review: an update on clinical, genetic and pathological aspects of frontotemporal lobar degenerations. Neuropathol Appl Neurobiol.

[3] Lui H, Zhang J, Makinson SR, Cahill MK, Kelley KW, Huang HY (2016). Progranulin deficiency promotes circuit-specific synaptic pruning by microglia via complement activation. Cell.

[4] Chitramuthu BP, Bennett HPJ, Bateman A (2017). Progranulin: a new avenue towards the understanding and treatment of neurodegenerative disease. Brain.

[5] Schmid CD, Sautkulis LN, Danielson PE, Cooper J, Hasel KW, Hilbush BS (2002). Heterogeneous expression of the triggering receptor expressed on myeloid cells-2 on adult murine microglia. J Neurochem.

[6] Hickman SE, Kingery ND, Ohsumi TK, Borowsky ML, Wang L, Means TK (2013). The microglial sensome revealed by direct RNA sequencing. Nat Neurosci.

[7] Takahashi K, Rochford CDP, Neumann H (2005). Clearance of apoptotic neurons without inflammation by microglial triggering receptor expressed on myeloid cells-2. J Exp Med.

[8] N’Diaye EN, Branda CS, Branda SS, Nevarez L, Colonna M, Lowell C (2009). TREM-2 (triggering receptor expressed on myeloid cells 2) is a phagocytic receptor for bacteria. J Cell Biol.

[9] Kleinberger G, Yamanishi Y, Suarez-Calvet M, Czirr E, Lohmann E, Cuyvers E (2014). TREM2 mutations implicated in neurodegeneration impair cell surface transport and phagocytosis. Sci Transl Med.

[10] Kleinberger G, Brendel M, Mracsko E, Wefers B, Groeneweg L, Xiang X (2017). The FTD-like syndrome causing TREM2 T66M mutation impairs microglia function, brain perfusion, and glucose metabolism. EMBO J.

[11] Xiang X, Werner G, Bohrmann B, Liesz A, Mazaheri F, Capell A (2016). TREM2 deficiency reduces the efficacy of immunotherapeutic amyloid clearance. EMBO Mol Med.

[12] Wang Y, Cella M, Mallinson K, Ulrich JD, Young KL, Robinette ML (2015). TREM2 lipid sensing sustains the microglial response in an Alzheimer’s disease model. Cell.

[13] Mazaheri F, Snaidero N, Kleinberger G, Madore C, Daria A, Werner G (2017). TREM2 deficiency impairs chemotaxis and microglial responses to neuronal injury. EMBO Rep.

[14] Paloneva J, Kestilä M, Wu J, Salminen A, Böhling T, Ruotsalainen V (2000). Loss-of-function mutations in TYROBP (DAP12) result in a presenile dementia with bone cysts. Nat Genet.

[15] Chouery E, Delague V, Bergougnoux A, Koussa S, Serre JL, Mégarbané A (2008). Mutations in TREM2 lead to pure early-onset dementia without bone cysts. Hum Mutat.

[16] Giraldo M, Lopera F, Siniard AL, Corneveaux JJ, Schrauwen I, Carvajal J (2013). Variants in triggering receptor expressed on myeloid cells 2 are associated with both behavioral variant frontotemporal lobar degeneration and Alzheimer’s disease. Neurobiol Aging.

[17] Guerreiro R, Bilgic B, Guven G, Brás J, Rohrer J, Lohmann E (2013). A novel compound heterozygous mutation in TREM2 found in a Turkish frontotemporal dementia-like family. Neurobiol Aging.

[18] Le Ber I, De Septenville A, Guerreiro R, Bras J, Camuzat A, Caroppo P (2014). Homozygous TREM2 mutation in a family with atypical frontotemporal dementia. Neurobiol Aging.

[19] Piccio L, Buonsanti C, Cella M, Tassi I, Schmidt RE, Fenoglio C (2008). Identification of soluble TREM-2 in the cerebrospinal fluid and its association with multiple sclerosis and CNS inflammation. Brain.

[20] Öhrfelt A, Axelsson M, Malmeström C, Novakova L, Heslegrave A, Blennow K (2016). Soluble TREM-2 in cerebrospinal fluid from patients with multiple sclerosis treated with natalizumab or mitoxantrone. Mult Scler J.

[21] Heslegrave A, Heywood W, Paterson R, Magdalinou N, Svensson J, Johansson P (2016). Increased cerebrospinal fluid soluble TREM2 concentration in Alzheimer’s disease. Mol Neurodegener.

[22] Piccio L, Deming Y, Del-Aquila JL, Ghezzi L, Holtzman DM, Fagan AM (2016). Cerebrospinal fluid soluble TREM2 is higher in Alzheimer disease and associated with mutation status. Acta Neuropathol.

[23] Henjum K, Almdahl IS, Årskog V, Minthon L, Hansson O, Fladby T (2016). Cerebrospinal fluid soluble TREM2 in aging and Alzheimer’s disease. Alzheimers Res Ther.

[24] Gispert JD, Suárez-Calvet M, Monté GC, Tucholka A, Falcon C, Rojas S (2016). Cerebrospinal fluid sTREM2 levels are associated with gray matter volume increases and reduced diffusivity in early Alzheimer’s disease. Alzheimers Dement.

[25] Suárez-Calvet M, Kleinberger G, Araque Caballero MÁ, Brendel M, Rominger A, Alcolea D (2016). sTREM2 cerebrospinal fluid levels are a potential biomarker for microglia activity in early-stage Alzheimer’s disease and associate with neuronal injury markers. EMBO Mol Med..

[26] Suárez-Calvet M, Araque Caballero MÁ, Kleinberger G, Bateman RJ, Fagan AM, Morris JC (2016). Early changes in CSF sTREM2 in dominantly inherited Alzheimer’s disease occur after amyloid deposition and neuronal injury. Sci Transl Med.

[27] Rascovsky K, Hodges JR, Knopman D, Mendez MF, Kramer JH, Neuhaus J (2011). Sensitivity of revised diagnostic criteria for the behavioural variant of frontotemporal dementia. Brain.

[28] Gorno-Tempini ML, Hillis AE, Weintraub S, Kertesz A, Mendez M, Cappa SF (2011). Classification of primary progressive aphasia and its variants. Neurology.

[29] Paterson RW, Heywood WE, Heslegrave AJ, Magdalinou NK, Andreasson U, Sirka E (2016). A targeted proteomic multiplex CSF assay identifies increased malate dehydrogenase and other neurodegenerative biomarkers in individuals with Alzheimer’s disease pathology. Transl Psychiatry.

[30] Meeter LH, Dopper EG, Jiskoot LC, Sanchez-Valle R, Graff C, Benussi L (2016). Neurofilament light chain: a biomarker for genetic frontotemporal dementia. Ann Clin Transl Neurol.

[31] Rohrer JD, Woollacott IOC, Dick KM, Brotherhood E, Gordon E, Fellows A (2016). Serum neurofilament light chain protein is a measure of disease intensity in frontotemporal dementia. Neurology.

[32] Bossù P, Salani F, Alberici A, Archetti S, Bellelli G, Galimberti D (2011). Loss of function mutations in the progranulin gene are related to pro-inflammatory cytokine dysregulation in frontotemporal lobar degeneration patients. J Neuroinflammation.

[33] Miller ZA, Rankin KP, Graff-Radford NR, Takada LT, Sturm VE, Cleveland CM (2013). TDP-43 frontotemporal lobar degeneration and autoimmune disease. J Neurol Neurosurg Psychiatry.

[34] Galimberti D, Bonsi R, Fenoglio C, Serpente M, Cioffi SMG, Fumagalli G (2015). Inflammatory molecules in frontotemporal dementia: cerebrospinal fluid signature of progranulin mutation carriers. Brain Behav Immun.

[35] Takahashi H, Klein ZA, Bhagat SM, Kaufman AC, Kostylev MA, Ikezu T (2017). Opposing effects of progranulin deficiency on amyloid and tau pathologies via microglial TYROBP network. Acta Neuropathol.

[36] Zhong L, Chen XF, Wang T, Wang Z, Liao C, Wang Z (2017). Soluble TREM2 induces inflammatory responses and enhances microglial survival. J Exp Med.

[37] Yin F, Banerjee R, Thomas B, Zhou P, Qian L, Jia T (2009). Exaggerated inflammation, impaired host defense, and neuropathology in progranulin-deficient mice. J Exp Med.

[38] Yin F, Dumont M, Banerjee R, Ma Y, Li H, Lin MT (2010). Behavioral deficits and progressive neuropathology in progranulin-deficient mice: a mouse model of frontotemporal dementia. FASEB J.

[39] Martens LH, Zhang J, Barmada SJ, Zhou P, Kamiya S, Sun B (2012). Progranulin deficiency promotes neuroinflammation and neuron loss following toxin-induced injury. J Clin Invest.

[40] Tanaka Y, Matsuwaki T, Yamanouchi K, Nishihara M (2013). Exacerbated inflammatory responses related to activated microglia after traumatic brain injury in progranulin-deficient mice. Neuroscience.

[41] Minami SS, Shen V, Le D, Krabbe G, Asgarov R, Perez-Celajes L (2015). Reducing inflammation and rescuing FTD-related behavioral deficits in progranulin-deficient mice with α7 nicotinic acetylcholine receptor agonists. Biochem Pharmacol.

[42] Piccio L, Buonsanti C, Mariani M, Cella M, Gilfillan S, Cross AH (2007). Blockade of TREM-2 exacerbates experimental autoimmune encephalomyelitis. Eur J Immunol.

[43] Sjögren M, Vanderstichele H, Agren H, Zachrisson O, Edsbagge M, Wikkelsø C (2001). Tau and Aβ42 in cerebrospinal fluid from healthy adults 21–93 years of age: establishment of reference values. Clin Chem.

[44] Paternicò D, Galluzzi S, Drago V, Bocchio-Chiavetto L, Zanardini R, Pedrini L (2012). Cerebrospinal fluid markers for Alzheimer’s disease in a cognitively healthy cohort of young and old adults. Alzheimers Dement.

[45] Forabosco P, Ramasamy A, Trabzuni D, Walker R, Smith C, Bras J (2013). Insights into TREM2 biology by network analysis of human brain gene expression data. Neurobiol Aging.

[46] Lue LF, Schmitz CT, Serrano G, Sue LI, Beach TG, Walker DG (2015). TREM2 protein expression changes correlate with Alzheimer’s disease neurodegenerative pathologies in post-mortem temporal cortices. Brain Pathol.

